# Acquired Bernard–Soulier-like syndrome due to a plasma-based inhibitor treated successfully with rituximab

**DOI:** 10.1016/j.rpth.2025.102727

**Published:** 2025-03-12

**Authors:** Lauren G. Banaszak, Paula A. Clark, Christopher G. Peterson, John Sheehan

**Affiliations:** 1 Department of Medicine, University of Wisconsin-Madison, Madison, Wisconsin, USA; 2 University of Wisconsin Health Special Coagulation Lab, Madison, Wisconsin, USA; 3 Department of Oncology-Hematology, Aspirus Health, Wausau, Wisconsin, USA; 4 University of Wisconsin Carbone Cancer Center, Madison, Wisconsin, USA

**Keywords:** autoantibody, Bernard-Soulier syndrome, bleeding, platelet aggregometry, platelet disorder

## Abstract

**Background:**

Bernard-Soulier syndrome (BSS) is an autosomal recessive disorder caused by deficient platelet glycoprotein Ib-IX-V expression resulting in abnormal bleeding, thrombocytopenia, giant platelets, and reduced platelet aggregation response to ristocetin that manifests in childhood. Acquired BSS is a rare disorder characterized by Bernard–Soulier (BS)-like platelet dysfunction in a patient without a history consistent with a bleeding disorder.

**Key Clinical Question:**

Can acquired BSS respond to immune-directed therapy?

**Clinical Approach:**

We describe a case of a 79-year-old man presenting with refractory epistaxis found to have an isolated BS-like platelet function defect due to a plasma-based inhibitor. He was treated with rituximab with immediate cessation of bleeding and normalization of platelet function studies.

**Conclusion:**

To our knowledge, this is the first case of acquired BS-like syndrome described in the absence of systemic illness due to a presumed autoantibody, and we report the successful use of rituximab for treatment of this rare disorder.

## Essentials

• Acquired BSS is a rare, acquired bleeding disorder.
• A patient presented with epistaxis and abnormal ristocetin-induced platelet aggregation.
• Mixing studies were consistent with the presence of a plasma-based inhibitor.
• Rituximab led to the resolution of symptoms, supporting its use in the treatment of acquired BSS.

## 1 Introduction

Primary hemostasis refers to the formation of a platelet clot in response to vascular injury. This entails a series of steps, including platelet adhesion to the subendothelial matrix at the site of vessel damage, platelet activation and release of granule contents, and fibrinogen-mediated platelet aggregation to form a stable platelet plug [1]. The binding of von Willebrand factor (VWF) in the subendothelial matrix and the glycoprotein (GP) Ib-IX receptor complex on the platelet surface is an important interaction that facilitates initial platelet adhesion. Reduced or abnormal expression of GPIb is the hallmark of Bernard–Soulier syndrome (BSS), an autosomal recessive disorder due to a genetic mutation in the *GP1BA*, *GP1BB*, or *GP9* gene [2]. BSS typically presents in childhood and is characterized by abnormal bleeding, moderate thrombocytopenia, and giant platelets [3]. Platelet aggregation studies usually show reduced or absent aggregation in response to ristocetin, a compound that enhances the binding of VWF to the GPIb complex *in vitro* and normal response to all other agonists, which are mediated by the binding of fibrinogen to the GP α_IIb_β_3_ complex [4]. Herein, we report a case of a 79-year-old man presenting with an acquired Bernard–Soulier (BS)-like syndrome due to a plasma-based inhibitor treated successfully with rituximab.

## 2 Case presentation

A 79-year-old man presented to the clinic for evaluation of recurrent epistaxis. Two months prior to presentation, the patient began to experience epistaxis involving the right nare on a daily basis. He was seen in the otolaryngology clinic, at which time his complete blood count and coagulation parameters were normal. No overt abnormalities were visualized on examination. He was treated with silver nitrate cautery to the right nare. Despite this, he continued to experience epistaxis, and further attempts at nasal packing and chemical cauterization were unsuccessful. One month prior to presentation, he was hospitalized for refractory nasal bleeding and was taken to the operating room for an endoscopic examination with electrocautery and nasal packing. Sphenopalatine artery ligation was attempted, but visualization was not possible due to the amount of bleeding. He was treated with oral tranexamic acid and platelet transfusions, which achieved hemostasis, and he was referred to hematology for coagulopathy evaluation.

The patient’s medical history was significant for diffuse large B-cell lymphoma diagnosed 30 years prior to presentation in remission following modified methotrexate, bleomycin, doxorubicin, cyclophosphamide, vincristine, dexamethasone chemotherapy; nodular lymphocyte-predominant Hodgkin’s lymphoma diagnosed 10 years prior to presentation in remission following rituximab, cyclophosphamide, vincristine, prednisone chemotherapy; autoimmune hemolytic anemia diagnosed concomitantly with Hodgkin’s lymphoma, which resolved after chemotherapy; immune thrombocytopenic purpura complicated by multiple relapses treated with steroids, splenectomy, intravenous immunoglobulin, and, most recently, rituximab 5 years prior to presentation; pulmonary embolism diagnosed incidentally during treatment for Hodgkin’s lymphoma; prior stroke with no residual neurologic deficits; diet-controlled type 2 diabetes; and chronic hepatitis B infection. His surgical history was notable for splenectomy, appendectomy, and cholecystectomy, which were not complicated by abnormal bleeding. Prior to this, he denied any history of excessive bleeding or bruising. There was no family history of bleeding disorders. He denied recent use of aspirin, anticoagulants, or herbal supplements and was taking only tranexamic acid at the time of evaluation.

Laboratory analysis showed a white blood cell count of 11.9 × 10^9^/L, hemoglobin level of 12.9 g/dL, platelet count of 168 × 10^9^/L, and normal kidney and liver function. The mean platelet volume was normal in both computed laboratory analysis and by direct visualization of the peripheral smear. Coagulation tests were notable for an international normalized ratio of 1.0, partial thromboplastin time of 28 seconds, thrombin time of 15.4 seconds, and fibrinogen level of 413 mg/dL. von Willebrand activity and antigen levels were both 241%, and factor VIII activity was 274%. Platelet aggregation was performed, which revealed normal aggregation and release in response to arachidonic acid (0.5 mM), adenosine diphosphate (10 μM), and collagen (2 μg/mL) and normal release in response to thrombin (1 U/mL; Figure A). However, the high-dose ristocetin (1.25 mg/mL) response was markedly attenuated, which was further reduced upon incubation (Figure B). There was no hypersensitivity noted in low-dose ristocetin (0.5 mg/mL; Figure B). Serum protein electrophoresis was normal. Computed tomography of the chest, abdomen, and pelvis did not show evidence of relapsed lymphoma or other abnormalities.

**Figure:**
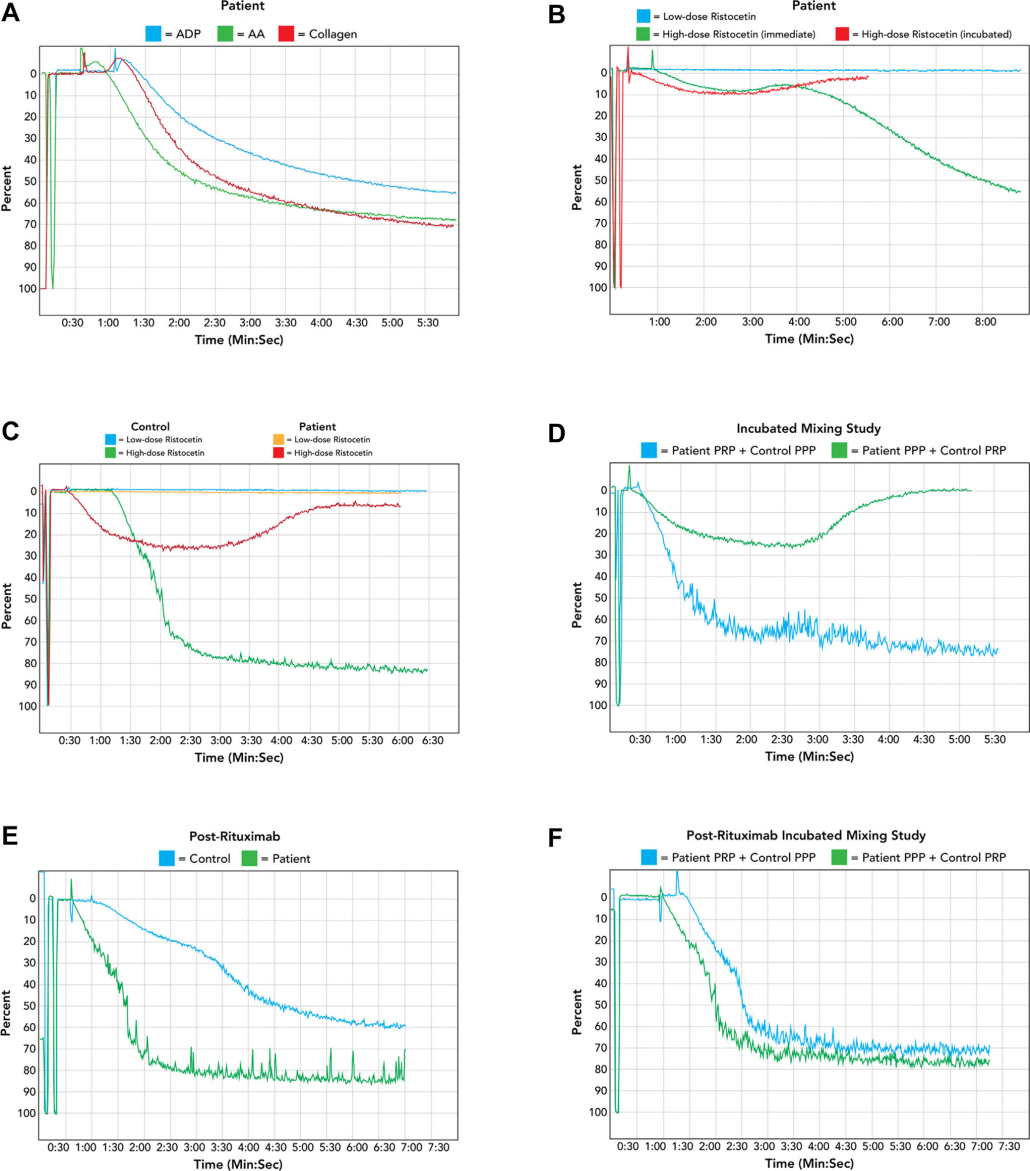
Platelet aggregation studies. (A) Normal platelet aggregation in response to adenosine diphosphate (ADP, 10 μM, blue curve), arachidonic acid (AA, 0.5 mM, green curve), and collagen (2 μg/mL, red curve). Response to thrombin (1 U/mL) was also normal (not shown). (B) Platelet aggregation in response to low-dose ristocetin (0.5 mg/mL, blue curve) and high-dose ristocetin (1.25 mg/mL) after immediate addition of an agonist (green curve) and after 1-hour incubation (red curve). The patient’s abnormal platelet response to high-dose ristocetin showed time dependency, with further attenuation of platelet aggregation observed after incubation. (C) There was no evidence of hypersensitivity to low-dose ristocetin (yellow curve), similar to the healthy control (blue curve). However, the patient’s platelet aggregation in response to high-dose ristocetin (red curve) was markedly attenuated compared with the healthy control (green curve). (D) Mixing of patient platelet-rich plasma (PRP) with control platelet-poor plasma (PPP) resulted in an initial delayed platelet aggregation response to high-dose ristocetin (data not shown) that subsequently normalized after incubation for 1 hour (blue curve). In contrast, the mixing of control PRP and patient PPP resulted in an initial normal aggregation response to high-dose ristocetin, followed by a markedly reduced magnitude of response and subsequent disaggregation after incubation for 1 hour (green curve). (E) Normal platelet aggregation response to high-dose ristocetin after treatment with rituximab (patient = green curve, control = blue curve). (F) Mixing of PRP + control PPP (blue curve) and patient PPP + control PRP (green curve) shows normal platelet aggregation in response to high-dose ristocetin with a 1-hour incubation after treatment with rituximab.

Repeat platelet aggregation studies confirmed the markedly defective patient response to high-dose ristocetin relative to a healthy donor control (Figure C). To evaluate for a plasma-based inhibitor, mixing studies were performed by the addition of platelet-poor plasma (PPP) to platelet-rich plasma (PRP) to attain a final platelet count of 250,000, followed by 1-hour incubation. Initially, PRP from the patient was mixed with PPP from a healthy donor control. Immediate addition of high-dose ristocetin resulted in a moderately attenuated platelet aggregation response (not shown) that subsequently normalized after incubation (Figure D). In contrast, when control PRP was mixed with patient PPP, the immediate addition of high-dose ristocetin resulted in normal aggregation (not shown), but the response was markedly reduced with subsequent disaggregation following incubation for 1 hour (Figure D). The time-dependent inhibition of the ristocetin response by patient PPP and resolution with control PPP suggested the presence of a plasma-based inhibitor.

The patient’s presentation and laboratory evaluation were suspicious for an acquired bleeding disorder most similar to BSS due to a plasma-based inhibitor, such as an autoantibody. Thus, he was urgently treated with rituximab 375 mg/m^2^ weekly for 4 weeks. One week after the completion of therapy, he had an immediate resolution of epistaxis. Platelet aggregation studies demonstrated normalization of the high-dose ristocetin response, and repeat incubated mixing studies demonstrated no evidence of inhibition (Figure E, F). An evaluation of platelet autoantibodies was performed after rituximab treatment, and this was negative for antibodies targeting GPIIB/IIIA, GPIB/IX, and GPIA/IIA. Over 3 years after the initial presentation, he remains in remission without evidence of recurrent bleeding.

## 3 Discussion

The term acquired or pseudo-BSS has been used to describe BS-like platelet dysfunction in the absence of a clinical history consistent with an inherited bleeding disorder. Acquired BSS is extremely rare, with only a few cases reported in the literature. Devine et al. [5] described a patient who developed severe thrombocytopenia and impaired ristocetin-induced aggregation of platelets after receiving procainamide therapy. She was found to have anti-GPIb autoantibodies, which were thought to be causative of an unusual form of drug-induced thrombocytopenia with a BS-type platelet defect. Berndt et al. [6] reported a 5-year-old girl with myelodysplastic syndrome complicated by severe thrombocytopenia associated with impaired platelet aggregation in response to ristocetin. It was hypothesized that the patient’s platelet abnormality was acquired as a consequence of her myelodysplastic syndrome, possibly due to a clonal chromosomal abnormality. BS-like platelet dysfunction has been described in 3 other pediatric patients with myeloid malignancies [7,8]. An acquired platelet defect resembling BSS has also been reported in patients with chronic autoimmune hepatitis, multiple myeloma, and an unspecified lymphoproliferative disorder [[9], [10], [11], [12]].

In this report, we add to the characterization of this rare disorder and to our knowledge present the first case of an apparently spontaneous acquired BS-like syndrome in the absence of active malignancy or preceding drug exposure. Our patient had no evidence of an inherited bleeding disorder, as evidenced by a lack of lifelong bleeding and undergoing multiple large surgeries without bleeding complications in the past. Platelet aggregation studies revealed a markedly defective response to high-dose ristocetin in the context of a normal platelet count, VWF antigen, and activity levels. Mixing studies demonstrated the time-dependent development of abnormal ristocetin responses in the presence of patient plasma and time-dependent improvement with control plasma, consistent with the presence of a plasma-based inhibitor. There was no evidence of active malignancy or infection based on history, laboratory tests, and imaging obtained at presentation. He was treated with rituximab with immediate cessation of bleeding and normalization of platelet function.

The plasma-based inhibitor, in this case, was hypothesized to be an autoantibody, which prompted treatment with rituximab. Though a platelet autoantibody was not identified, testing occurred after rituximab therapy, given the urgent need for treatment, which may have facilitated autoantibody clearance. The patient’s dramatic response to rituximab and his extensive history of abnormal humoral immunity is also supportive of an antibody-mediated process. Of the few cases of acquired BSS reported in the literature, 3 demonstrated transmission of the impaired ristocetin response with plasma, and 2 identified platelet-specific antibodies [5,9,10,12]. Thus, it seems likely that an autoantibody is responsible for this patient’s presentation.

Interestingly, our patient did not demonstrate thrombocytopenia during his clinical course. Antibody-induced platelet dysfunction in the context of a normal platelet count has only rarely been reported [12]. Autoantibodies directed against GPIb are well-described in immune-mediated thrombocytopenia [13]. However, platelet function is typically normal. This suggests that the target binding site of the autoantibody in acquired BSS is a distinct region of the GPIb-IX receptor complex that impairs function but does not result in accelerated platelet clearance or perhaps an entirely novel antigen involved in ristocetin-induced platelet aggregation. Indeed, the patient with acquired BSS reported by Stricker et al. [12] was found to have an autoantibody targeting a 210,000 molecular weight protein present on the platelet surface postulated to facilitate platelet and VWF interaction.

In summary, we report a patient without evidence of an inherited bleeding disorder or active malignancy who presented with refractory epistaxis, normal platelet count, and an isolated defect of ristocetin-induced platelet aggregation. Further studies revealed the presence of a plasma-based inhibitor suggestive of an autoantibody-mediated acquired BS-like syndrome. He was treated with rituximab with a resolution of his bleeding symptoms and normalization of platelet aggregometry studies. More studies are needed in order to elucidate the pathophysiology of acquired BSS, including autoantibody targets, which may shed light on novel mechanisms of normal platelet physiology and primary hemostasis.
